# Silver diamine fluoride for the management of dental caries in children in primary dental care: protocol for a feasibility study

**DOI:** 10.1186/s40814-024-01519-y

**Published:** 2024-06-24

**Authors:** Laura Timms, Helen Rodd, Chris Deery, Paul Brocklehurst, Zoe Marshman

**Affiliations:** 1 School of Clinical Dentistry, University of Sheffield, Sheffield, UK; 2 Dental Public Health, Primary Care Division, Public Health Wales, 10 Llys Castan Parc Menai, Bangor, Wales UK

**Keywords:** Feasibility study, Silver diamine fluoride, Paediatric dentistry, Cariology, Caries management, Minimally invasive caries management, Primary care

## Abstract

**Background:**

Dental caries remains a significant problem in England, affecting 11% of 3-year-olds and 23% of 5-year-olds. While current approaches have been extensively investigated, their ability to (1) control pain and infection; (2) prevent hospital admissions, and (3) be implemented within the National Health Service (NHS) contractual arrangements, remains unsatisfactory. Silver diamine fluoride (SDF) is an alternative, non-invasive approach that has proven efficacy in arresting caries progression in primary teeth, principally from studies conducted outside of Europe*.* Its use in primary dental care in the UK is limited, despite the acknowledged need.

The clinical and cost-effectiveness of SDF has not been compared to usual care in the UK. Before a pragmatic randomised controlled trial (RCT) can be conducted to compare SDF to usual care for caries management in young children, there are several uncertainties that require investigation. This study aims to establish whether such an RCT is feasible.

**Methods:**

This mixed-method parallel design study is a feasibility study with an embedded process evaluation, to compare SDF with usual treatment in primary dental care in the UK. It will be individually randomised, with 13 dentists and therapists, in 8 different dental primary care sites with a sample size of 80 child participants aged 1–8 years old. The aim will be to recruit ten participants per site with equal arm allocation. Follow-up will be for 1 year. The study will inform whether an RCT is feasible by resolving several key uncertainties. The acceptability and implementation of SDF and the research processes will be explored. Patient and Public Involvement and Engagement representatives will be involved throughout recruitment and retention strategies, participant documentation, analysis, engagement and dissemination.

**Discussion:**

The ability to conduct an RCT will be evaluated. If feasible, this RCT has the potential to evaluate the effectiveness of a non-invasive approach for the management of untreated caries in young children. A feasibility study also offers the opportunity to consider factors associated with the implementation of SDF at an early stage through a process evaluation that will inform the definitive trial and an implementation strategy for SDF by identifying relevant barriers and facilitators.

**Trial registration:**

ClinicalTrials.gov identifier: NCT06092151. Date: 19/10/2023.

**Supplementary Information:**

The online version contains supplementary material available at 10.1186/s40814-024-01519-y.

## Background

Despite national evidence-based preventative guidance, English data suggest that dental caries affects 11% of 3-year-olds and 23% of 5-year-olds [1–3]. It is well established that dental caries places a significant burden on children, their parents, the health service and society [4–8].

Notwithstanding the impact on children and the extensive research in the area, the clinical management of young children with caries in their primary teeth remains a challenge. There is a spectrum of options available for caries management in children which range from topical treatments that are minimally invasive to those that are more conventional such as fillings under local anaesthetic. The desired outcome of treatment is to halt the progression of caries preventing it from causing pain or infection before the primary tooth exfoliates. If caries progresses, primary teeth usually require removal, and for some young children, this necessitates multiple dental extractions under general anaesthetic (GA). The cost of hospital admissions for extractions for children in England was £50 million in 2015–2016 [9].

The Care Index is a measure used in epidemiological surveys that demonstrates the proportion of carious teeth that are filled or removed; this figure is only 14% for 5-year-olds in England [3, 10]. There are myriad reasons why this figure is so low [11, 12] including uncertainty over treatment protocols and increasing numbers of children being referred to hospital services [13–15]. General dental practitioners have questioned the merits of guideline-recommended management, and research echoes this finding, demonstrating that this advised treatment is infrequently carried out [16–18]. A recent British study sought to address this uncertainty over management protocols[ however, there was a high divergence from allocated treatments, further confirming clinician uncertainty and variable practice [11]. Caries progressed in many participants, with 42.7% experiencing pain or infection over a median follow-up period of 33.8 months [11].

Although caries management approaches have been extensively investigated, evidence for their ability to (1) control pain and infection; (2) prevent hospital admissions, and (3) be implemented within the current National Health Service (NHS) contractual arrangements remains lacking.

An alternative intervention for caries management is silver diamine fluoride (SDF) [19]. Studies, mostly conducted in Asia and South America, have reported that SDF has efficacy in arresting caries in primary dentition [19]. A meta-analysis found that SDF outperformed two active treatments for caries arrest with a risk ratio of 66% [20]. However, adoption of SDF is limited in the UK. Although previous studies have been conducted in different countries, populations and health settings, there is no fundamental reason to believe that SDF would not be efficacious in a UK child population. Nonetheless, key aspects of these trials have important implications for their generalisability to the UK. The main differences are (1) the comparators used in the control arms are inconsistent with national UK guidance; (2) the prevalence and severity of dental caries; (3) access to fluoride and prevention regimes, and; (4) the majority of trials included in systematic reviews were school-based, with none undertaken in a setting comparable to UK primary dental care settings with their unique funding arrangements. These differences (to the current research described in this protocol) limit the translation of their findings to a UK primary dental care context.

It, therefore, remains unknown whether SDF would be more effective at controlling caries in children’s primary teeth than alternatives that are recommended in widely used and evidence-based guidance, and whether this different approach would be transferable to UK primary dental care.

As such, a pragmatic randomised controlled trial (RCT) in the UK, in a primary care setting, comparing SDF to usual care is required to establish whether SDF can impact the unmet need of managing caries in the primary dentition. There are several uncertainties that need to be addressed ahead of this RCT that will be achieved by this feasibility study. An important consideration is the acceptability of SDF. While SDF is simple for children to manage as a clinical technique, it discolours carious lesions black. Acceptance of this side effect has therefore been highlighted as a concern by dentists in the UK [21]. A recent systematic review, involving eight international studies, found that parental acceptance was highly variable (ranging from 0 to 100%) [22]. Therefore, it is also paramount that acceptability and determinants of implementation of SDF in the UK are explored from clinician and patient/parent perspectives. This feasibility study will address uncertainties to consider the feasibility and allow planning of an RCT which will seek to meet the burden of dental caries for children, their families and the NHS. It will also involve an embedded process evaluation to consider the acceptability of SDF, and the research processes in order to optimise them ahead of a trial should it be deemed feasible.

## Methods/study design

### Aim

To establish whether conducting an RCT to compare SDF to usual care for the treatment of caries in children’s primary teeth is feasible.

### Objectives

1. Establish which suitable primary and secondary outcome measures
2. Calculate the variability of the chosen primary outcome measure
3. Establish whether recruitment, randomisation and retention of participants are feasible
4. Establish the ability to recruit and retain dental professionals
5. Establish the rate of dentists’ adherence to the study protocol
6. Establish the ability to complete an economic evaluation
7. Explore the acceptability and feasibility of treatment with SDF for children, families, dentists and key stakeholders
8. Explore the acceptability and feasibility of the research processes for children, families, dentists and key stakeholders

### Design

A pragmatic RCT is required to investigate the effectiveness of SDF compared to usual care in the UK for the treatment of caries in children’s primary teeth. Ahead of this RCT, there are several uncertainties that require clarification to assess the feasibility of the trial:

1. Primary and secondary outcome measures.
2. Variability of primary outcome measure.
3. Recruitment and retention of participants.
4. Recruitment and retention of dentists.
5. Deviation from treatment allocation and SDF treatment protocol.
6. Ability to complete economic evaluation.
7. Acceptability and feasibility of treatment with SDF for children, families, dentists and key stakeholders.
8. Acceptability and feasibility of the research processes for children, families, dentists and key stakeholders.

This protocol describes this feasibility study (work package one) and embedded theoretically informed process evaluation (work package two) that will seek to address these uncertainties.

### Setting

The feasibility study will be based on eight different NHS primary dental care sites (general dental practices and community clinics). These are located across Yorkshire and the Humber and the East Midlands where the prevalence and severity of caries is higher than the national average. This setting provides external validity supporting translation to practice, as primary care is where the majority of children with dental caries receive care [9].

The practices will be from different areas in terms of deprivation, location (urban or rural), with different NHS contracts, practice sizes and skill mix to reflect NHS primary care nationally. Dentists and dental therapists will collect data and provide the intervention and comparator.

Qualitative interviews will take place at a private location chosen by the participant, for example, the dental practice, the University of Sheffield or the participant’s home or school.

### Participant characteristics

The study population will be children aged 1–8 years, who have caries into dentine in at least one of their primary teeth. This age group is appropriate as there is a high proportion of children with both caries and lack of restorations or preformed metal crowns as recommended in current guidance. Where treatment is not carried out or fails and extraction is required, these young children usually require general anaesthesia for this treatment. Therefore, they are a key cohort where an alternative solution to treatment is a salient issue.

The parent and/or carer must be able to give informed consent with the support of an interpreter if necessary. Patients whose treatment requires special consideration for example due to a medical condition or a dental anomaly will be excluded. Children with caries that have spread to cause pain or infection, or are involved in the pulp of the tooth will not be included as the SDF treatment would be contra-indicated.

The work package two involves a sub-sample of the parents of these children, who must be able to take part in a qualitative interview, with the support of an interpreter or researcher speaking their first language if required.

Work packages one and two will also involve data collection from dental professionals involved in the study on the feasibility and acceptability of SDF and the research processes. They must be dental professionals or non-clinical staff working in a practice involved in the feasibility study. National and regional stakeholders will also be invited to interview.

### Sample size

Data from work package one will be used to calculate the required sample size of the RCT. Sim and Lewis suggest a sample size of at least 50 for the feasibility study is required to determine the variability of the primary outcome measure [23]. The sample size of the feasibility study must also allow estimations of other important parameters of uncertainty [24]. Estimates for these parameters are based on previous research in a similar participant group in NHS primary dental care [11] and 80 child participants would give the ability to estimate:

1. A willingness to be randomised of 81% within a 95% confidence interval of ± 8.6%
2. A drop-out rate of 33% within a 95% confidence interval of ± 10.2%
3. A deviation from the treatment allocation rate of 24% within a 95% confidence interval of ± 9.4%

Therefore, 80 is an appropriate sample size to meet the study objectives. It will allow for a heterogeneous sub-sample to be recruited for qualitative interviews (work package 2).

The sample will be made up of the following:

1. A purposive sample of child and parent/carer dyads from the SDF group, and from the control group in work package one. Recruitment will continue until data saturation is reached. Based on previous studies it is estimated that 25–30 children and 25–30 parents/carers will be required [25, 26]. Participants will be sampled to include those from living in areas of deprivation, with differing levels of caries experience, age and ethnicity.
2. A purposive sample of dental staff involved in the study and treating patients. Recruitment will continue until data saturation is reached with an anticipated sample size of 20–30 [27].
3. Policy makers and national and regional stakeholders with relevant experience regarding the implementation and acceptability of SDF, will be recruited through existing networks. The anticipated sample size is 5–8.

### Recruitment

Practice dental records will be screened for potentially eligible patients attending from the study start date and onwards for 3 months, who will be sent a postal letter and/or email with participant information leaflets for children and carers designed with the study patient and public involvement and engagement (PPIE) panel. Information will be translated and interpreters used as necessary. Subsequently, eligibility will be confirmed by a dentist when children attend their check-ups. Given the young age of the children, assent will be sought but where parents/carers provide informed consent this will be accepted for children to participate in the study.

Parents/carers will be asked to indicate whether they would be prepared to take part in an interview with their child at a later date. A purposive subsample of those who consent to be contacted will subsequently be contacted with new participant information leaflets and informed consent completed. The sample will include those of a range of ages, sex, ethnicities, dental practices, treatment received and socioeconomic status.

At the first treatment visit, participants will be randomised and baseline assessments made. Randomisation is a computer generated by the sealed envelope system, the participant must have consented before this is undertaken. At this visit, their first allocated treatment will be provided. Allocation will determine treatment for all teeth meeting the selection criteria. If the family wishes and they had the information ahead of their appointment, consent (Appendix 1) and treatment may take place at the same visit. Sponsor protocols for data confidentiality will be followed throughout.

### Intervention

SDF is a topical treatment that has efficacy in arresting the progression of caries in the primary dentition. Soft tissues are protected and SDF is applied topically to the teeth with a small brush, with a maximum of one capsule applied per visit. Within the study, the application of SDF will follow the British Society of Paediatric Dentistry (BSPD) standard operating procedure (SOP) [28]. SDF will be applied at the initial treatment visit and at 6 months recall as per national and international guidance. If the caries is still active at an earlier review, it will be re-applied at this point.

### Comparator

For the purposes of this feasibility study, based on previous research and discussions with primary dental care dentists, usual care is variable within primary dental care. The best practice is as described in the Scottish Dental Clinical Effectiveness Programme Guidelines for the management of caries in primary dentition [29]. For posterior teeth, this would involve the use of preformed metal crown placement using the Hall Technique or placing a minimally invasive composite resin restoration. In terms of the anterior teeth, minimally invasive composite restorations are advised as first-line treatment. However, it is acknowledged that treatment in primary care does not always follow these guidelines, and there is uncertainty over the best management option [11, 16, 17]. As such dentists are free to provide usual care for participants in the way that they feel is in the child’s best interest given the clinical scenario; however, training will be provided outlining the guideline recommended care. This variation will form part of the assessment of the feasibility of delivering the RCT as planned in terms of the dentist’s treatment provided in the usual care comparator arm.

### Visits and data collection

This is a parallel design mixed-method study. Quantitative and qualitative data will be collected simultaneously throughout the feasibility study and process evaluation [30, 31]. Data will be analysed separately and then considered together when interpreting the findings.

All participants will be of high caries risk status and thus will be reviewed 3-monthly in accordance with NICE guidance [32, 33]. The dentists and dental therapists will complete a case report form that will collect candidate primary and secondary outcome measures at baseline and 3-month recalls. There is no core outcome set for caries trials and the heterogeneity of outcome measures has been highlighted in previous systematic reviews of SDF [20, 34]. The appropriate measures will be investigated to capture both clinical and patient-reported outcomes.

The following candidate outcome measures have been chosen based on those employed and validated in previous UK studies in primary dental care, discussion with research supervisors who are experienced in caries trials and trials with children, and the PPIE group and their ability to meet the research question of the definitive trial [11, 35, 36].

Candidate primary outcome measures:

- The success of treatment using the International Caries Detection and Assessment System (ICDAS) [37].
- Occurrence or report of pain or infection. The Pulpal involvement, Ulceration, Fistula, Abscess (PUFA) index will be used to measure advanced caries [38].

Candidate secondary outcome measures:

Clinician-reported the following:

- Referral to secondary care for either caries management in general or specifically care under general anaesthesia.
- Completion of courses of treatment.
- The number of appointments taken for completion of a course of treatment.
- Adherence to intervention protocol or usual care guidelines.
- Adverse effect(s).
- Appointment length and number of units of dental activity claimed

Patient-reported data will be collected using questionnaires including measures of:

- Child and parental/carer reported experience of treatment.
- Parental/carer reported oral health-related quality of life.
- Familial cost related to dental treatment.

At baseline and completion, the following data will be collected:

- Parent/carer-reported measure of their child’s oral health-related quality of life using the 16-item Parent-Caregiver Perceptions Questionnaire [39].
- Parent/carer reported oral health behaviours.

At appointments where treatment is carried out the following will be collected:

- Child and parent/carer-reported experience of treatment using a 3-point pictorial face scale, and a 5-point scale, respectively, utilised in the Filling Children’s Teeth: Indicated Or Not (FicTION) study [11].

A parameter of uncertainty is the rate of dental health professionals’ adherence to the treatment allocation and any deviation from treatment protocol. Data will be collected pertaining to these uncertainties through the case report form (CRF) to establish, what treatment was carried out and whether this was consistent with the participants’ allocation. Further information will be collected to include whether the SDF treatment protocol was used, was the visit schedule and data were collected as planned with reasons given where this was not the case. Data will be entered into SPSS by LT.

Qualitative interviews will also include an exploration of any deviations from the study protocol and SOP.

Cost data will be collected at treatment visits, and at 3-month recalls from both the CRF and parent/carer questionnaires, this will include data from an NHS healthcare perspective and from a patient perspective.

Data will be collected to review whether recruitment, randomisation and retention of participants are feasible through screening and recruitment logs, and a record of participant attendance along with feedback from participants who left the study where possible.

The ability to recruit and retain dental professionals is also a key uncertainty, data will be collected to record the number of invited dentists, those who wished to participate and those who subsequently had the capacity to participate. Feedback will be sought from those on their decision to participate or decided not to remain in the study.

In order to address both objectives 7 and 8 of the study; explore the acceptability and feasibility of treatment with SDF, and the research processes for children, families, dentists and key stakeholders, using both qualitative and quantitative methods. Quantitative data collection pertaining to reach, dose and fidelity of SDF delivery will be collected through the CRF. This will include whether SDF was implemented as per the treatment protocol in terms of quantity and fidelity to the protocol and whether the treatment allocation was deviated. Child-reported and parent/carer-reported experiences of the treatment will be collected when treatment is carried out.

The topic guides for semi-structured qualitative interviews include aspects related to the:

- Acceptability of SDF is based on the seven constructs of Sekhon’s acceptability framework [40].
- Acceptability and feasibility of the research processes (including recruitment, retention and adherence to treatment allocation).
- Implementation of SDF into routine practice based on elements of the consolidated framework of implementation research (CFIR) [41].

Different topic guides will be used for children, parents/carers, dental practice teams and national/local stakeholders.

### Data analysis

Quantitative data will be analysed using simple descriptive statistics as recommended in the literature [42, 43]. An assessment will be made of data quality. Estimation of confidence intervals will be carried out for objectives 3 and 5, acknowledging the limitation of sample size. These descriptive statistics will be used to consider the feasibility of conducting a trial. If a definitive RCT is deemed feasible this will be planned according to the findings of this study.

The primary outcome measures will be assessed based on data quality and qualitative findings. There will be a discussion with the advisory group and the study PPIE panel as to which best meets the research question when planning a subsequent trial. RCTs are often underpowered, the feasibility study will provide a more accurate estimate of the variability of the chosen primary outcome measure to allow sample size calculation for the definitive trial [44]. The variability of the chosen primary outcome measure for the definitive RCT will be calculated.

Qualitative data will be analysed using framework analysis, using a combination of deductive and inductive coding adhering to the following stages [45]:

- 1. Transcription of data.
- 2. Familiarisation with data.
- 3. Coding and development of a working framework for analysis.
- 4. Application of the developed framework to the dataset.
- 5. Charting of data into the framework matrix.
- 6. Interpretation of findings.

Analysis will be reviewed with the study PPIE panel, supervisory team and advisory group (including general dental practitioners), to ensure views are represented.

The CFIR will be used as an organising framework for interview aspects related to implementation, with Sekhon’s framework guiding the analysis of acceptability [40, 46].

## Discussion

Practical issues surrounding the delivery of this study include the challenges of carrying out research generally and in dental care specifically [47]. This includes recruitment, training and calibration of dental professionals, recruitment and retention of participants, adherence to protocol and allocation and data quality. These potential issues justify the need for a feasibility study and have informed the uncertainties that require exploration and therefore the objectives of this study. The participants in this study are likely to include those who are acknowledged to be underserved in research [47]. To attempt to make this study more inclusive of these participants, advice from PPIE representatives has been followed. This includes the use of translated documents, interpreters for qualitative research, information in multi-media formats and delivering the study in primary care.

Young children are participants in the study and assent will be sought from those who are able to provide it, in addition to obtaining consent from their parents. Some of the PPIE representatives included young children with caries and their parents who advised on how best to share information with other children. Following this guidance, children co-produced a multi-media information video to support information sharing with other children in a format that they can understand (https://sites.google.com/d/1NC7JvNoe8hZNl90oUhfTtoH05T4QSWIg/p/11Wi50XRyVFwHgVp5SGly-K7fAwdOEtaR/edit?pli=1). This format will be used in order to disseminate study information to the public following its conclusion.

The anticipated timeline is participant recruitment from July to December 2023. Following this, participants will be followed up for 1-year after their treatment. Throughout this period qualitative interviews will be completed. The anticipated end date is December 2024. Any protocol changes will be communicated through IRAS and email to participating sites.

Findings from this completed feasibility study will help to determine whether it is possible to deliver a future RCT and how best to design this, taking into account the views and experiences of primary dental care professionals and patients. As this is an early-stage feasibility study, formal progression criteria are not included. Results for each parameter will be reviewed together with qualitative data to determine whether delivering a definitive trial will be feasible. Depending on the number of changes required, an internal pilot with formal progression criteria may be undertaken.

This study will inform whether SDF as a treatment is acceptable to children, parents and dental teams and whether it is possible to implement it successfully. Factors affecting implementation will be identified through the process evaluation to support the delivery of SDF in primary care dental practice.

This study will be used in order to determine the primary outcome measure, consequent sample size required and subsequently the number of practices needed to successfully deliver a definitive trial. Findings will inform how best to support the recruitment and retention of both practices and participants. This will allow efficient and effective recruitment and determine training packages related to research processes and treatments. It will identify how much ongoing support from the central trial team is required and in what format. The most appropriate data collection methods will be designed based on the findings from the study in tandem with the PPI panel.

### Supplementary Information

Supplementary Material 1.

## Data Availability

Anonymised data will be available on request.

## Abbreviations

SDF: Silver diamine fluoride
NHS: National Health Service
RCT: Randomised controlled trial
UK: United Kingdom
GA: General anaesthetic
BSPD: British Society of Paediatric Dentistry
ICDAS: International Caries Detection and Assessment System
PUFA: The Pulpal involvement, Ulceration, Fistula, Abscess
SOP: Standard operating procedure
CRF: Case report form
CFIR: Consolidated framework of implementation research
PPIE: Patient and public involvement and engagement
