# Supplementary material for: Silver diamine fluoride for the management of dental caries in children in primary dental care: protocol for a feasibility study
Source: Pilot Feasibility Stud. 2024 Jun 24;10:95. doi: 10.1186/s40814-024-01519-y (PMC11194932; doi:10.1186/s40814-024-01519-y)

Appendix 1

Consent form for feasibility study


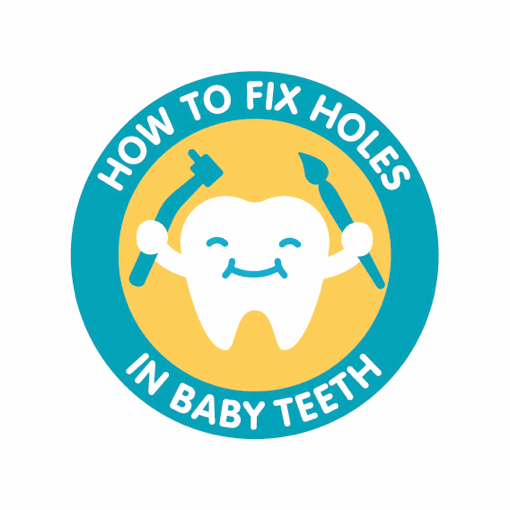

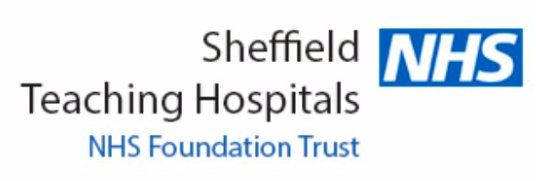

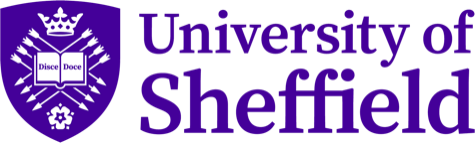


**Parent / Carer Consent Form**

**How to fix holes in baby teeth?: Feasibility Study**

Participant ID :

If you would like your child to participate in the study and agree with each statement, please initial the boxes corresponding to each statement and sign below.

1. I confirm that I have read and understand the information sheet for the

study, version 2.1 dated 04/05/2023. My child and I have had the

opportunity to consider the information, ask questions, and have had

these answered satisfactorily.

1. I have had chance to ask questions about the dental treatments involved

and these have been answered satisfactorily

1. I understand that the participation of my child is voluntary and

that I am free to withdraw at any time without giving any reason and

without there being any negative consequences. In addition, should I or

my child not wish to answer any particular question or questions in the questionnaires, I or my child are free to decline.

**Please turn to the next page**

1. I understand that personal information that identifies me and my child will

be collected from me for use in the study, this will be through an information

log and this consent form. It will be stored securely.

1. I understand the treatment my child receives will be decided at random. I understand the dentist remains free to treat my child based on what they

consider to be in their best interests.

1. I give permission for the research team to have access to the anonymised

responses of me and my child, and to the information collected by the

dental team about my child. I understand that neither me or my child will

be identifiable in the results.

1. I am aware and agree that information obtained during the study will be

analysed and treated as confidential and nothing identifying me or my

child personally will be made publicly available or shared.

1. I am aware that anonymised information about my child may be published.
2. I know how to contact the research team if I need to.
3. I understand that data collected during the study may be looked at by

individuals from The University of Sheffield, regulatory authorities or from

the NHS Trust where it is relevant to my child taking part in this research.

1. I give consent to be contacted about me and my child participating in

an interview about my child’s treatment (optional).

1. I consent to my child taking part in this study

**Please turn to the next page**

________________________

Name of participant (child)

________________________ ________________ ____________________

Name of parent/carer of Date Signature

participant (child)

_________________________ ________________ ____________________

Name of person taking consent Date Signature

**Thank you for taking part.**

Original: dental team (patient records). Copies: 1 for signatory; 1 for researcher (site file)


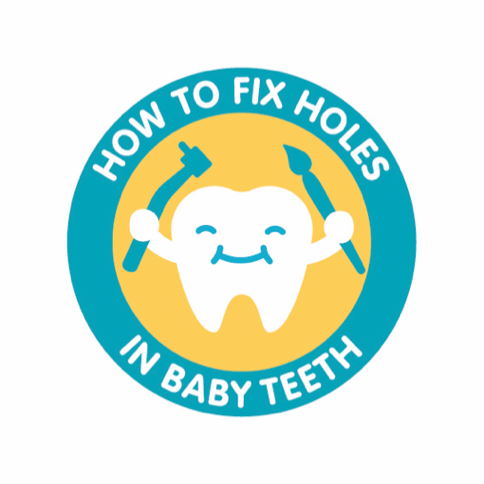

Supplement: Supplementary file 1 — Supplementary Material 1. [file 40814_2024_1519_MOESM1_ESM.docx]
